# ST/HR variables in firefighter exercise ECG – relation to ischemic heart disease

**DOI:** 10.14814/phy2.13968

**Published:** 2019-01-27

**Authors:** Anna Carlén, Eva Nylander, Meriam Åström Aneq, Mikael Gustafsson

**Affiliations:** ^1^ Department of Clinical Physiology Linköping University Linköping Sweden; ^2^ Department of Medical and Health Sciences Linköping University Linköping Sweden

**Keywords:** Electrocardiography, low risk, ST depression, ST/HR variables

## Abstract

Exercise electrocardiography (ExECG) is regularly performed by Swedish firefighters by law. Heart rate‐corrected analysis of ST segment variables (ST/HR) has shown improved prediction of ischemic heart disease (IHD) compared to ST depression alone. This has not previously been extensively studied in asymptomatic persons with a low probability of IHD. We therefore evaluated the predictive performance of ST/HR analysis in firefighter ExECG. ExECG was studied in 521 male firefighters. During 8.4 ± 2.1 years, 2.3% (*n *= 12) were verified with IHD by catheterization or myocardial scintigraphy (age 51.5 ± 5.5 years) and were compared with firefighters without imaging proof of IHD (44.2 ± 10.1 years). The predictive value of ST depression, ST/HR index, ST/HR slope, and area and rotation of the ST/HR loop was calculated as age‐adjusted odds ratios (OR), in 10 ECG leads. Predictive accuracy was analyzed with receiver operating characteristics (ROC) analysis. ST/HR index ≤−1.6 *μ*V/bpm and ST/HR slope ≤−2.4 *μ*V/bpm were associated with increased IHD risk in three individual leads (all OR > 1.0, *P *< 0.05). ST/HR loop area lower than the fifth percentile of non‐IHD subjects indicated IHD risk in V4, V5, aVF, II, and –aVR (*P* < 0.05). ST depression ≤−0.1 mV was associated with IHD only in V4 (OR, 9.6, CI, 2.3‐40.0). ROC analysis of each of these variables yielded areas under the curve of 0.72 or lower for all variables and leads. Clockwise‐rotated ST/HR loops was associated with increased risk in most leads compared to counterclockwise rotation. The limited clinical value of ExECG in low‐risk populations was emphasized, but if performed, ST/HR analysis should probably be given more importance.

## Introduction

Exercise testing in firefighters is used to ensure sufficient physical capacity to manage the strenuous tasks of firefighting, and to identify individuals with possible undetected heart disease. The mandatory evaluation of smoke‐diving firefighters in Sweden consists of periodic standard bicycle ergometer exercise ECG (ExECG) and treadmill‐based submaximal fitness tests, used for risk assessment and exercise capacity respectively (Swedish Work Environment Authority, 2005).

Exercise‐induced ST segment depression is the most established ExECG marker for IHD but a weak predictor in low‐risk groups (Gianrossi et al. 1989). In general, screening of low‐risk individuals is therefore discouraged (Lauer et al. 2005; Greenland et al. 2010). Improved approaches for ExECG analysis beyond ST depression are hence sought after. The ST/HR slope represents the peak rate of ST segment changes as a function of heart rate (HR) during end‐exercise, and was initially suggested to evaluate the severity of IHD (Elamin et al. 1980). The mathematically simpler ST/HR index then evolved, dividing overall ST depression with HR increase during exercise (Detrano et al. 1986). Subsequently, the recovery phase was added to the analysis, yielding ST/HR recovery loop characterization (Okin et al. 1989) and area of the ST/HR hysteresis (Lehtinen et al. 1996). ST/HR analysis has in several studies improved both the diagnostic and prognostic ability of the ExECG, also in asymptomatic populations (Kligfield et al. 1989; Okin et al. 1997). In the Framingham offspring study of asymptomatic men and women, the relative risk of coronary events was 1.9 when either the ST/HR index or the ST/HR loop was abnormal, and when both were positive the relative risk was 3.6, whereas standard ST segment depression criteria were not predictive of new events. (Okin et al. 1991).

In people with physically demanding occupations, absence of disease is important, but test interpretation may be problematic and lead to both unnecessary and costly diagnostic procedures. The underlying research question of this study was therefore: can the increase in sensitivity and specificity conferred by an in depth ECG analysis including ST deviation in relation to heart rate during both exercise and recovery – and not only ST level at maximum work – improve ExECG performance enough to make it a useful tool also in low prevalence populations?

### Aim

The aim of this study was to investigate ExECG in a cohort of firefighters, with special focus on the predictive performance of ST depression and in particular ST/HR variables, in relation to IHD.

## Material and Methods

### Study population

All male firefighters in a Swedish county, who had performed at least one bicycle ergometer exercise test between January 2004 and December 2010, were initially eligible for the study (*n *= 751). Informed consent was obtained from 550 subjects to allow for access to the county medical records, which were examined until June 2015 in search for history of cardiac disease and cardiac imaging studies.

According to exclusion criteria that could hamper the interpretation of the ST/HR analysis, subjects with an implanted pacemaker or a history of myocardial infarction were excluded, as well as ExECG tests with insufficient signal quality (see below), bundle branch block, exercise induced arrhythmia, and left ventricular hypertrophy with secondary ST‐T changes, thereby excluding a total of 5.3% (29 subjects).

### Cycle ergometer test

Cycle ergometer exercise tests were carried out as incremental ramp tests with a continuous increase by 1 W every 3 sec until exhaustion, either starting with 6 min at 200 W or 250 W, followed by immediate conversion to ramp test, or a ramp protocol during the entire test.

All ExECG tests were registered with CardioPerfect (Welch Allyn Inc, Skaneateles Falls, NY). Limb electrodes were placed in the Mason–Likar modification of the standard 12‐lead ECG. ST level measured 60 msec after the J‐point (ST60) and heart rate (HR) were stored every 15 sec during exercise and up to at least 3 min of recovery. Systolic blood pressure was measured every 3 min during cycling.

Firefighters with exercise test abnormalities including ST depression, arrhythmias and – although infrequent – symptoms, were routinely referred to the cardiology department for further evaluation.

### Processing of exercise ECG data

A structured query language (SQL) script interrogated the exercise ECG database to extract data into a study database. The HR and ST data acquired were analyzed for signal noise and in doubtful cases the ECGs were manually checked. Affected leads or tests were discarded from further calculations when inappropriate for analysis. Leads V1 and aVL were excluded from all analyses, as suggested by Okin et al. (1989) and adopted by several investigators, because of insufficient diagnostic information (Kligfield et al. 1989; Lehtinen et al. 1996; Viik et al. 1997; Svensbergh et al. 2004; Kronander et al. 2005, 2011). ST60 data were filtered with a three‐point moving median, to reduce signal noise.

### Exercise ECG variables

Baseline heart rate (HR_start_) sitting on the bicycle and peak HR (HR_peak_) were calculated as the median of the three first recorded heart beats at sitting rest and the three last beats in the exercise phase respectively. Median values were used to avoid sporadic artifacts. Target HR (HR_pred_) was defined as ≥85% of age‐predicted maximum HR. (Tanaka et al. 2001).

**Figure 1:**
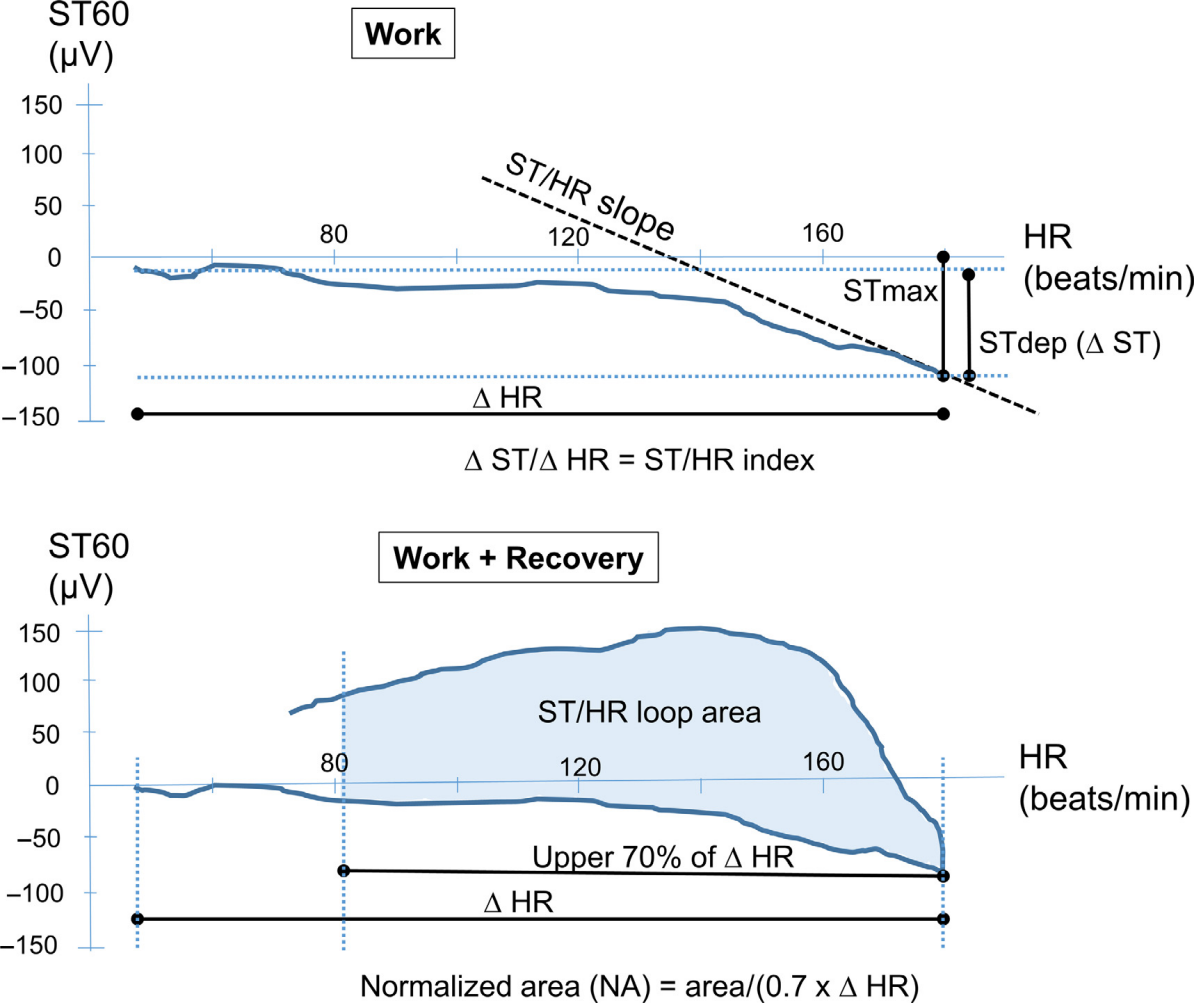
ST and ST/HR values from the exercise ECG. Schematic illustration of ST60 values (*x* axis) plotted against HR (*y* axis) during exercise and recovery of the exercise ECG. ST_max_, ST_dep_, ST/HR index, and ST/HR slope are derived from the work phase (upper panel) while work and recovery phases combined generates direction and area of the ST/HR loop (lower panel). ST_dep_ represents additional negative ST deflection, excluding any ST depression at baseline. The ST/HR slope is a regression line with a significant correlation coefficient, representing the end of the work phase (at least the final four ST/HR pairs). The area of the ST/HR loop is normalized to HR, by dividing the included area by the corresponding HR increase. HR, heart rate; ST_dep_, ST depression; NA, normalized area.

Peak ST deviation (ST_max_) and slope of the ST segment (ST_slope_) were calculated as the median value of the last three measurements at peak workload. ST depression (ST_dep_), ST/HR index and ST/HR slope were analyzed in leads where ST_max_ was negative. ST_dep_ was defined at peak workload, as the difference between ST_max_ and ST_start_, or if ST_start_ was positive, as difference from the isoelectric PQ level (Okin et al. 1990). The ST/HR index reflected ST_dep_ at peak workload divided by the exercise‐induced HR change (Detrano et al. 1986). The ST/HR slope (*μ*V/bpm) was calculated by linear regression analysis, beginning with the final four ST/HR data points during exercise, and progressively including previous ST/HR points (Okin and Kligfield 1995). See Figure 1. The steepest slope with significant correlation coefficient (*P *< 0.05) was accepted as the ST/HR slope for that lead. ST/HR index ≤−1.6 *μ*V/bpm and ST/HR slope ≤−2.4 *μ*V/bpm were considered suggestive of ischemia (Kligfield et al. 1989).

**Figure 2:**
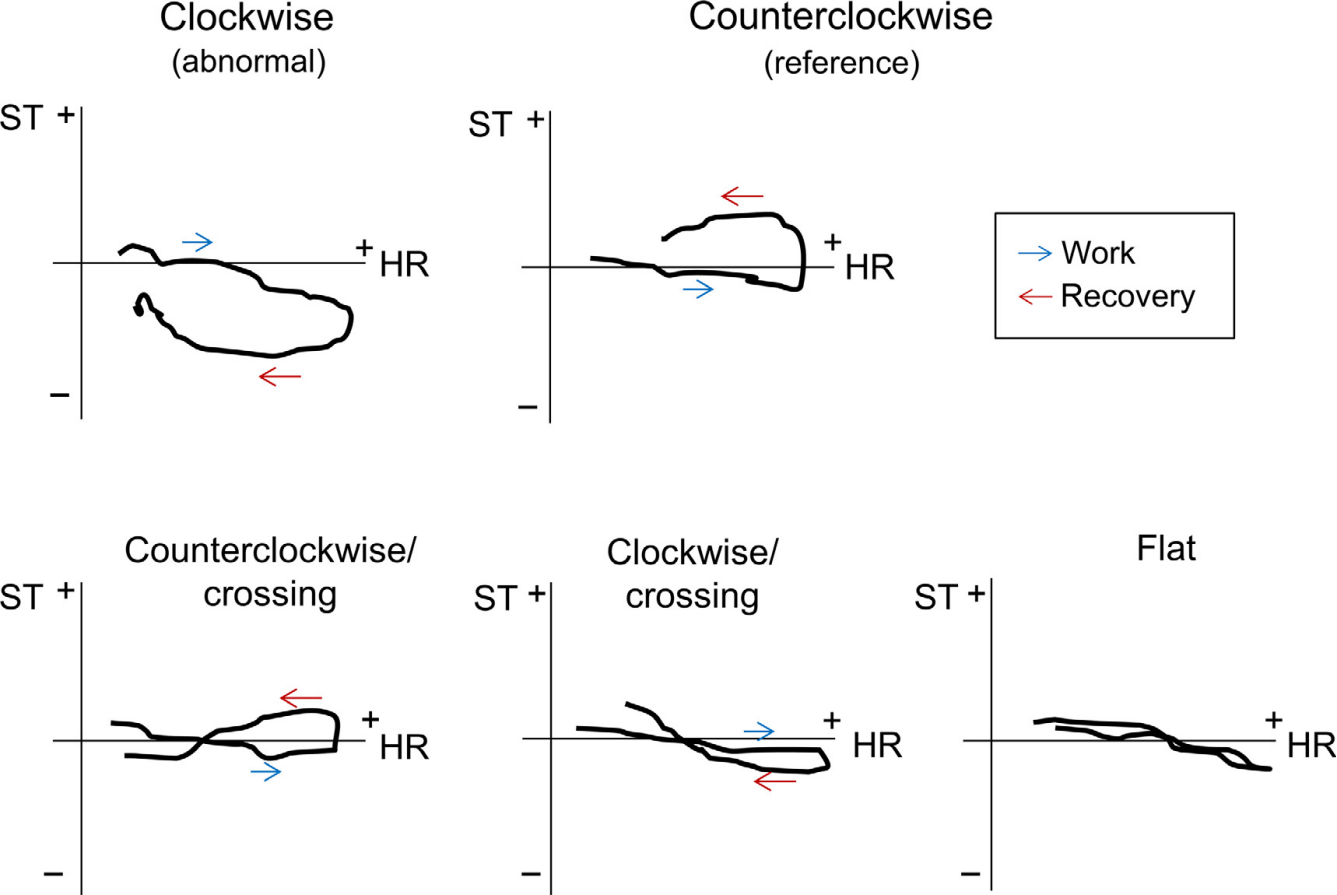
Typical patterns of the ST/HR recovery loop. Schematic illustrations of typical ST/HR loop rotation patterns. ST60 values (*x* axis) plotted against HR (*y* axis) during exercise and recovery of the exercise ECG test. Blue arrows indicate work phase data and red arrows indicate recovery. Open loops were either rotated counterclockwise or clockwise (upper panel). Intermediate patterns included initial counterclockwise rotation with subsequent crossing (lower left), counterclockwise rotation with subsequent crossing (lower center), and flat loops with minimal area and indefinite rotation (lower right).

An ST/HR loop for each lead was generated by plotting exercise and recovery ST60 values on the *y*‐axis and HR values on the *x*‐axis, covering the HR span of the first 3 min of recovery. We defined ST/HR loop rotation by comparing the corresponding ST levels of the work phase and the recovery phase for each recorded HR registration. If ST levels in the resting phase were numerically larger than during the work phase, the loop was determined to be rotating counterclockwise (positive comparison), and vice versa for negative comparisons (clockwise rotation). “Crossing” loop pattern (i.e., taking the form of a lying 8) was evaluated by splitting the HR span of the loop and comparing the direction of rotation between the two halves. Loops with small distance between the exercise and recovery ST levels were classified as flat See Figure 2. Loops that did not fit in to any of those definitions were not analyzed.

The normalized area (NA) of the ST/HR loop was calculated from ST60 data for each lead. As described by Svensbergh et al. (2004), the upper 70% of the HR increase was used in the calculation and the area was divided by the included HR span (Fig. 1). The fifth percentile of the NA was identified for each lead in those who did not develop IHD during follow‐up and those values were applied as cutoffs in the regression analysis.

### Statistics

SPSS statistical software (IBM SPSS Statistics for Windows, Version 24.0. Armonk, NY) was used. Independent samples *t*‐test was used for comparison of continuous data and risk estimates were calculated by binary logistic regression analysis. Exp(B) from the regression analysis was presented as odds ratio (OR) with 95% confidence interval (CI). All ORs were adjusted for age. Receiver operating characteristics (ROC) curves, including calculation of the area under the curve (AUC), analyzed the predictive accuracy of different ST and ST/HR parameters in relation to IHD. In the ROC analysis, ST/HR index and ST/HR slope were calculated for all individuals and leads, independent of STmax value.

Significance level for all analyses was set at *P *< 0.05.

### Ethical approval

This study was conducted according to the principles of the Declaration of Helsinki and it had Regional Ethic Review Board approval.

## Results

We studied ExECG from 521 male firefighters. Final workload (*P*_max_) was 279 ± 39 W and HR_peak_ was 172 ± 12 beats/min, corresponding to 97% of age‐predicted maximal HR.

During 8.4 ± 2.1 years of follow‐up, 11% had gone through at least one cardiac imaging study according to the county medical records. Twelve subjects had significant coronary artery stenosis or exercise‐induced ischemia verified by coronary angiography or myocardial scintigraphy (IHD, *n *= 12). These subjects were compared to the 509 subjects who had either negative (*n *= 35), inconclusive/nonsignificant findings (*n *= 8) or no available imaging studies (*n *= 466) during the entire follow‐up.

In the IHD group, the nearest test prior to a positive imaging study was selected, on average 2.4 ± 2.1 years before the positive imaging study and 8.1 ± 2.0 years before the end of the follow‐up period. Among non‐IHD subjects, tests were selected to match as similar follow‐up time as possible (mean 7.5 ± 1.5 years). Mean time between ExECG and diagnostic test in the non‐IHD group was 2.1 ± 3.0 years.

IHD subjects were significantly older at the time of the analyzed test than non‐IHD subjects, but neither weight, height, nor BMI differed (Table 1).

**Table 1:** Characteristics of the study sample and basic test data

	IHD, *n *= 12		Non‐IHD, *n *= 509		*P*
	Mean	SD	Mean	SD	
Age, years	51	6	44	10	0.001
Height, m	1.79	0.05	181	0.06	0.315
Weight, kg	90	18	86	11	0.382
BMI	28.2	4.9	26.2	3.0	0.199
HR_start_, beats/min	82	13	80	14	0.552
HR_peak_, beats/min	166	20	172	12	0.280
HR_pred_, %	95		97		0.656
BP_peak_, mmHg	216	27	202	22	0.025

BMI, body mass index; BP, blood pressure; HR, heart rate; HR_pred_, peak heart rate in relation to predicted maximal heart rate; IHD, ischemic heart disease.

**Table 2:** Test results and risk for ischemic heart disease in different ECG leads

	ST depression and ST/HR variables												ST/HR loop characterization									
	ST_dep_ ≤−0.1 mV			ST/HR index ≤−1.6 *μ*V/bpm			ST/HR slope ≤−2.4 *μ*V/bpm			NA ≤5th percentile			CCW (ref)	Clockwise			Counterclockwise/Crossing			Flat		
Lead	*n*	OR	CI	*n*	OR	CI	*n*	OR	CI	*n*	OR	CI	*n*	*n*	OR	CI	*n*	OR	CI	*n*	OR	CI
V2	2	–	–	1	–	–	4	0	0.0–	26	1.3	0.2–10.9	469	2	–	–	15	4.7	0.9–24.6	8	0	0.0–
V3	1	–	–	1	–	–	5	6.9	0.7–68.8	27	3.3	0.7–16.2	477	4	12.0*	1.1–130.3	12	2.6	0.3–23.1	1	–	–
V4	18	9.6**	2.3–40.0	9	27.5***	5.6–134.5	37	5.1*	1.4–18.4	28	5.2*	1.3–21.5	467	4	12.0*	1.1–132.7	21	1.4	0.2–12.5	0	–	–
V5	55	3.0	0.8–10.9	29	4.3*	1.0–17.4	79	2.7	0.8–9.3	28	5.3*	1.3–21.9	462	4	41.1**	5.0–340.4	28	1.5	0.2–12.7	3	13.9*	1.0–193.4
V6	64	2.6	0.7–9.3	29	4.0	1.0–16.7	86	2.5	0.7–8.5	27	3.5	0.7–17.4	454	6	26.0**	3.9–174.5	33	2.3	0.4–11.7	3	0	0.0–
I	2	–	–	0	–	–	16	0	0.0–	27	4.5	0.9–22.3	412	4	43.0**	3.0–620.6	22	0	0.0–	41	1.6	0.3–8.4
−aVR	8	4.1	0.5–37.2	3	18.7*	1.6–224.1	32	2.7	0.6–13.1	28	6.0*	1.5–24.2	460	3	57.2**	3.5–932.4	14	0	0.0–	14	5.3	1.0–29.4
II	33	2.3	0.5–11.0	16	5.0	1.0–25.7	50	5.7**	1.7–19.2	29	8.5**	2.3–31.1	459	4	22.7*	1.9–268.4	26	5.5*	1.3–23.6	6	0	0.0–
aVF	38	2.1	0.4–10.3	15	2.4	0.3–20.1	53	3.9*	1.1–13.5	28	6.3*	1.6–25.4	441	10	6.6	0.7–62.1	23	6.4*	1.5–27.1	18	0	0.0–
III	53	1.6	0.3–7.5	23	1.9	0.2–15.2	77	2.6	0.7–8.9	26	2.1	0.3–17.5	396	29	5.0	0.9–26.5	21	5.4*	1.0–29.2	27	1.7	0.2–15.6
Max ST_dep_	105	1.5	0.4–5.2	58	3.0	0.8–10.8	136	1.4	0.4–4.8	26	2.5	0.3–20.9										
Peak	105	1.5	0. 4–5.2	57	3.1	0.9–11.1	159	1.6	0.5–5.3	27	5.0	1.0–25.6										

Risk expressed as age‐adjusted odds ratio (OR) with 95% confidence interval (CI). *N* represents number of subjects with positive finding for each analyzed variable and lead. The NA fifth percentile represents lead‐specific cutoff values based on distribution among non‐IHD subjects. Counterclockwise ST/HR loop rotation (CCW) was used as normal reference for other loop rotation patterns. Clockwise/crossing loops had OR* *= 0 in all leads (not shown). Not calculated for groups with *n *≤ 2.

HR, heart rate; Max ST_dep_, lead with maximum ST_dep_; NA, normalized area of the ST/HR loop; Peak, lead with largest value of the assessed parameter; ST_dep_, ST depression.

*,** and *** stand for significance at <0.05, <0.01, and <0.001 levels respectively.

### ST depression

Significant peak exercise ST depression (ST_dep_ ≤−0.1 mV), was found in at least one lead in 20% of the study participants. During follow‐up, IHD was objectively verified in 4% of the participants . Any lead ST_dep_ ≤−0.1 mV was not associated with increased risk for IHD (OR, 1.5, CI, 0.4–5.2). ST_dep_ ≤−0.1 mV in two or more leads was found in 15% of the cohort and 10% had ST_dep_ ≤−0.1 mV in two or more chest leads. Neither of the groups had significantly increased age‐adjusted risk for IHD.

**Figure 3:**
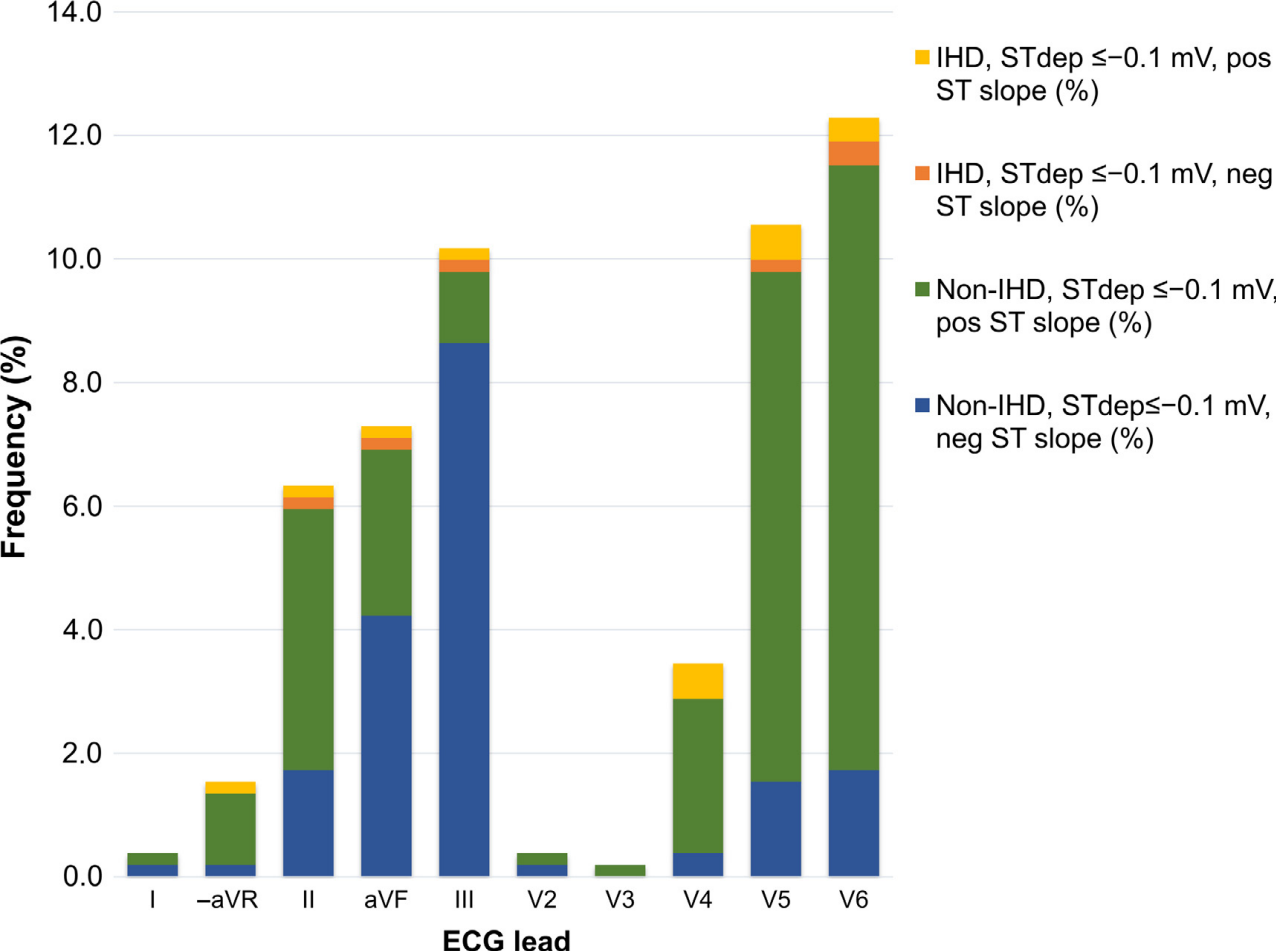
ST depression in different leads. Frequency (%) of exercise‐induced ST depression (ST_dep_) ≤−0.1 mV in different leads. Fraction calculated within entire study sample. Bars subdivided by the direction of the ST segment slope and finding of ischemic heart disease (IHD) during follow‐up.

ST_dep_ ≤−0.1 mV was most common in lateral and inferior leads, with >10% prevalence in leads V5, V6, and III. Upsloping ST depression dominated in the precordial leads, whereas in extremity leads, a negative slope was more common (Fig. 3). Lead‐specific analysis showed that ST_dep_ ≤−0.1 mV, independent of slope, was associated with increased risk for IHD only in V4 (Table 2). Downsloping ST_dep_ ≤−0.1 mV was associated with significantly increased OR for IHD only in V6 (OR, 9.1, CI, 1.7–49.0).

### ST/HR index

ST/HR index ≤−1.6 *μ*V/bpm in the precordial lead with the most prominent ST depression was associated with increased risk for IHD (OR, 4.5, CI, 1.2–16.8), which was not the case for the extremity lead with the largest ST depression (OR, 3.5, CI, 0.7–17.4), nor the lead with maximum ST depression or the lead with the lowest ST/HR index. Specific leads with increased ST/HR index risk estimates were V4, V5, and –aVR (OR, 27.5, 4.3, and 18.7 respectively, *P *< 0.05). See Table 2 for complete results.

### ST/HR slope

Future risk for IHD was increased in tests where the maximal extremity lead ST depression had an ST/HR slope ≤−2.4 *μ*V/bpm (OR, 4.4, CI, 1.4–14.0), as opposed to ST/HR slope data from the lead with the maximal precordial ST depression (OR, 2.2, CI, 0.6–7.5), maximal overall ST depression, or the lead with the lowest ST/HR slope (Table 2). ST/HR slope below the cutoff was associated with increased risk in V4, aVF, and II (OR, 5.1, 3.9, and 5.7 respectively, *P *< 0.05) (Table 2).

### ST/HR loop characteristics

In all leads, counterclockwise rotation of the ST/HR loop dominated (76–92% prevalence in different leads) and was used as a normal reference in comparison with other loop patterns (Okin et al. 1989; Lehtinen et al. 1996).

The IHD risk in clockwise‐rotated loops was higher compared to reference in all leads, except in aVF and III (V2 not analyzed because of too few cases). The highest risk estimate was found in –aVR, where three subjects had a clockwise‐rotated loop, OR, 57.2 (CI, 3.5–932.4, *P *< 0.01).

The intermediate loops were subcategorized into clockwise/crossing, counterclockwise/crossing, and flat. In total, 219 loops (4.2% of all leads in all subjects) could not be characterized because of either equal number of recovery ST/HR data points under and above the exercise level in one of the loop halves, missing recovery ST/HR data points, or sporadic outliers.

In no lead, clockwise/crossing loops were associated with greater risk for IHD compared to reference. On the contrary, counterclockwise/crossing loops had significantly increased risk for IHD in aVF (OR, 6.4), II (OR, 5.5), and III (OR, 5.4), as well as did flat loops in V5 (OR, 13.9) (Table 2).

### Area of the ST/HR loop

For each ST/HR loop, the normalized area (NA) was calculated. The lead‐specific cutoff values for NA, defined as lower than the fifth percentile among non‐IHD subjects, were spread closely around zero in extremity leads (between −18 and +10 *μ*V), and in precordial leads slightly higher (range 10–35 *μ*V). An NA below the cutoff was associated with an increased risk for IHD, significantly so in V4, V5, aVF, II, and –aVR (all with OR, 5.2–8.5, *P *< 0.05). The cutoff for the lead with the maximal ST depression was −14 *μ*V (OR for IHD, 2.5, CI, 0.3–20.9) and for the lead with the lowest NA the cutoff was −36 *μ*V (OR for IHD, 5.0, CI, 1.0–25.6) (Table 2).

### Predictive ability in different variables and leads

**Figure 4:**
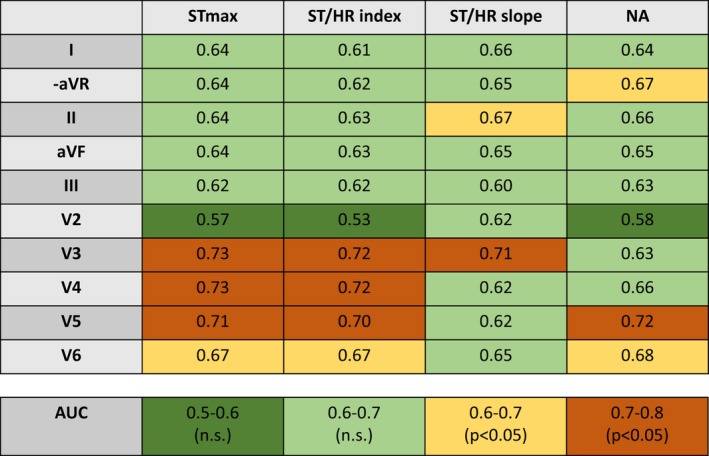
Receiver operating characteristics (ROC) curves.

The predictive ability of the studied ST/HR variables and ST_max_ was determined by calculating the area under the curve (AUC) in ROC analyses (Fig. 4). This was performed for all ST/HR indices and ST/HR slopes, irrespective of positive or negative ST_max_. Since ST depression was only present in a minority of the population, the variable ST_dep,_ that would be represented by 0 in most cases, was not included in the ROC analysis and instead replaced by ST_max_.

In the extremity leads, cutoffs that combined high sensitivity and specificity were generally not available in this population, for none of the studied variables. Only ST/HR slope in lead II and NA in –aVR had areas under the curves (AUC) that were significantly different from 0.5 (AUC 0.67 in both cases). The precordial leads V5–V6 had AUCs above 0.5 for ST_max_, ST/HR index, and NA, range 0.67–0.72 with the highest AUC found for NA in V5. Lead V3 had AUC values of 0.71–0.73 for all variables except NA and lead V4 had AUC values of 0.72 and 0.73 for ST/HR index and ST_max_ respectively.

## Discussion

The low predictive value of exercise‐induced ST depression for identification of IHD in asymptomatic subjects has limited its use in the clinician′s toolbox. The use of more complex variables in the ST/HR analysis has improved the diagnostic and prognostic ability in some asymptomatic populations (Kligfield et al. 1989; Okin et al. 1991, 1997). We therefore aimed to evaluate their usefulness in an occupational screening setting.

### ST depression

Exercise‐induced ST depression in asymptomatic subjects has been reported to be associated with an increased risk for cardiovascular events (Chou et al. 2011). In our study, every five firefighter had ST_dep_ ≤−0.1 mV in at least one lead by the end of exercise, but neither that nor an ST depression in two chest leads was associated with increased risk for IHD. Only in V4 was an ST depression which was associated with disease. Thus, ST depression, when found, was in most cases false positive and of limited value for prediction of IHD.

### ST/HR index

ST/HR index below cutoff tended to generate higher risk estimates than ST_dep_ ≤−0.1 mV in all leads, but with significant association with disease only in V4, V5, and –aVR as well as from the precordial lead with the greatest ST depression. ST/HR index has previously shown to improve prediction of IHD compared to ST depression alone, for example in asymptomatic men and women in the Framingham Offspring Study (analyzing the lead with the most prominent ST depression) (Okin et al. 1991). Our results suggest that while analysis of ST/HR index seems superior to ST depression alone, there are still major limitations indicated by the low AUC in the ROC analysis.

### ST/HR slope

The ST/HR slope was initially used to quantify myocardial ischemia in patients with IHD (Okin and Kligfield 1995) but it has also been proposed for diagnostic purposes. In a clinical population where more than one‐third of patients were verified with IHD, ST/HR slope had slightly higher sensitivity for detection of IHD than both ST/HR index and ST depression (Kronander et al. 2010). In our population, with presumably asymptomatic subjects and a low event rate during follow‐up, ST/HR slope below cutoff was only associated with IHD in three individual leads (V4, aVF, and II) as well as in the extremity lead with the deepest ST depression. However, the possibility to compare risk estimates between variables is limited because of the widespread confidence intervals.

### ST/HR loop characteristics

We found clockwise‐oriented loops to be associated with increased risk for IHD in the majority of leads (Table 2). The sensitivity of clockwise‐oriented loops in a previous study was 93% among those with IHD proven by angiography (Okin et al. 1989). Such a high sensitivity may have been reached because more than half of that study population had effort‐related chest pain and therefore a substantially higher pretest probability of disease than the subjects in this cohort, who were more similar to the clinically healthy reference group. Still, our findings suggest that clockwise‐oriented loops should be considered pathological in asymptomatic subjects because of the association to IHD across most leads.

There is no established method for how to characterize the ST/HR loop. Originally it was described as a dichotomous variable with either of two directions, defined by the ST deviation at 1 min of recovery (Okin et al. 1989). Later, the analysis was extended to ≥3 min of recovery. With more frequent data points, more complex loop configurations were seen and categorization included intermediate patterns (Suurküla et al. 2001). We found that crossing loop patterns were associated with IHD if the initial rotation was counterclockwise, consistent with previous research (Johansen et al. 2008). On the contrary, loops starting to rotate clockwise but with a subsequent crossing were not associated with IHD in any of the leads. This indicates that end‐exercise ST changes with rapid normalization as HR decreases are generally not pathological in low‐risk populations, which may help to identify an ST depression as false positive. Both counterclockwise/crossing and flat loops were in some leads associated with IHD and could be observed as abnormal in asymptomatic subjects, but not directly suggestive of IHD.

Criteria for computerized distinction between flat and crossing loops with little distance between the exercise and recovery curves may affect the results. Further methodological research may be valuable to address this topic.

### Area of the ST/HR loop

ST/HR hysteresis has been shown to be a better prognostic marker of myocardial infarction than both ST depression and other ST/HR variables (Kronander et al. 2011). The normalized area (NA) of the ST/HR loop reflects the behavior of the ST segment in the HR domain during work and recovery combined and can, just like the loop rotation, theoretically distinguish between physiological ST depression with rapid normalization and ischemic ST depression that recovers slowly. The now frequently used cutoffs for both ST/HR index and ST/HR slope were originally defined by identifying the fifth percentile value among subjects without IHD. We used the same approach to identify lead‐specific cutoffs, that is partition values above which 95% of the healthy subjects were found. In a previous study of NA, performed in a population with about 50% IHD prevalence, the extremity and precordial leads with the most negative NAs were combined, which detected IHD with 89% sensitivity and 77% specificity (Svensbergh et al. 2004). In our study where cutoffs were similar, sensitivity and specificity were lower. Still, a small ST/HR loop area was better at indicating risk for IHD than ST depression, especially in extremity leads (Table 2).

### Predictive ability of different variables

ROC analysis revealed overall low AUC values, not exceeding 0.72, which indicates barely acceptable possibilities for a variable to adequately distinguish between true and false positive test results. A perfect test combining 100% sensitivity and 100% specificity generates an ROC curve with an area under the curve of 1.0. Compromising either sensitivity or specificity alters the rate of true versus false results.

ST_max_ and ST/HR index had AUCs significantly different from 0.5 in four precordial leads each, whereas for ST/HR slope and NA fewer leads had acceptable discriminating capacity.

As reflected by the low AUC values, the ability to identify true cases of ischemic heart disease within a large asymptomatic cohort, as in the present study, was limited, both for ST_max_ as well as for the ST/HR variables.

### Lead selection

In the previous research, analyses have often included one or two ECG leads per test, based on either the most prominent ST depression or the most abnormal value of the studied parameter. If maximal ST depression would have been used in this study, precordial and extremity leads would have been represented in most cases by V6 and III, which were not the leads with the highest risk estimates for IHD for any of the studied variables. Instead, when all leads were analyzed, important leads with present but not maximal ST depression were found to be better determinants of IHD risk, especially including V4. In leads V2 and I, risk estimates did not reach significance for any of the studied variables. Also ROC analysis revealed low discriminating capacity in the extremity leads. The different utility of different leads has been shown before (Viik et al. 1997). In summary, we discourage choosing the lead with the most prominent ST depression for ST/HR analysis.

### Limitations

In this cohort, comprising active firefighters, only 2.3% developed manifest IHD during follow‐up. With such a low event rate, the predictive value of any screening test can be expected to be low, but was not known for ST/HR variables, that have previously been proven superior to ST depression in population studies with a similar event rate (Okin et al. 1991). The low incidence of IHD may contribute to nonsignificant findings in the regression analysis; however, a cohort of more than 500 firefighters is still a relevant sample for exploration of the positive predictive value of ExECG in a low‐risk population, also when the more complex parameters describing ST/HR relationships are applied. The low number of positive cases implicated generally very wide confidence intervals, limiting the comparison between variables.

The mean time interval between ExECG and positive cardiac imaging was 2.4 years. Potentially, IHD can develop in such time period and some IHD cases could be true negative at the time of the ExECG. However, the screening procedure is aimed to detect subclinical disease in an asymptomatic population in which imaging is not routinely performed, and the mean time interval between the ExECG and the IHD diagnosis in this study was similar to the frequency of the screening tests. The design of the evaluation and the results obtained, are thus relevant for the population and the circumstances.

The study was restricted to male firefighters and the findings cannot be generalized to females.

## Conclusions

ST analysis when combined with HR data during the work phase (ST/HR index and ST/HR slope) tended to assess IHD risk better than ST depression alone, especially so for precordial lead ST/HR index and extremity lead ST/HR slope. When adding the recovery phase to the analysis (ST/HR loop rotation and loop area) an increase in the number of predictive leads was seen. The added value was, however, still low.

Despite analyzing multiple variables from the ExECG, none combined high sensitivity and high specificity, which implies that both false‐negative and false‐positive test results remain an issue in this type of population. Our results reinforce the question of the clinical relevance of performing repeated exercise tests in firefighters or similar populations. However, in case of performing exercise tests in individuals with low pretest probability, ST/HR variables should be given more importance.

## Conflict of Interest

The authors report no conflict of interest.
